# A blinded study using laser induced endogenous fluorescence spectroscopy to differentiate ex vivo spine tumor, healthy muscle, and healthy bone

**DOI:** 10.1038/s41598-023-50995-4

**Published:** 2024-01-22

**Authors:** Jacob Sperber, Tanner J. Zachem, Ravi Prakash, Edwin Owolo, Kent Yamamoto, Annee D. Nguyen, Harrison Hockenberry, Weston A. Ross, James E. Herndon, Patrick J. Codd, C. Rory Goodwin

**Affiliations:** 1 Department of Neurosurgery, Duke University School of Medicine, Durham, USA; 2 Department of Mechanical Engineering and Materials Science, Duke University, Durham, USA; 3 Department of Biostatistics & Bioinformatics, Duke University School of Medicine, Durham, USA; 4 Duke Cancer Institute, Duke University Medical Center, 200 Trent Drive DUMC 3807, Durham, NC 27710 USA

**Keywords:** Biotechnology, Cancer, Cell biology, Oncology, Engineering

## Abstract

Ten patients undergoing surgical resection for spinal tumors were selected. Samples of tumor, muscle, and bone were resected, de-identified by the treating surgeon, and then scanned with the TumorID technology ex vivo. This study investigates whether TumorID technology is able to differentiate three different human clinical fresh tissue specimens: spine tumor, normal muscle, and normal bone. The TumorID technology utilizes a 405 nm excitation laser to target endogenous fluorophores, thereby allowing for the detection of tissue based on emission spectra. Metabolic profiles of tumor and healthy tissue vary, namely NADH (bound and free emission peak, respectively: 487 nm, 501 nm) and FAD (emission peak: 544) are endogenous fluorophores with distinct concentrations in tumor and healthy tissue. Emission spectra analyzed consisted of 74 scans of spine tumor, 150 scans of healthy normal bone, and 111 scans of healthy normal muscle. An excitation wavelength of 405 nm was used to obtain emission spectra from tissue as previously described. Emission spectra consisted of approximately 1400 wavelength intensity pairs between 450 and 750 nm. Kruskal–Wallis tests were conducted comparing AUC distributions for each treatment group, α = 0.05. Spectral signatures varied amongst the three different tissue types. All pairwise comparisons among tissues for Free NADH were statistically significant (Tumor vs. Muscle: p = 0.0006, Tumor vs. Bone: p < 0.0001, Bone vs. Muscle: p = 0.0357). The overall comparison of tissues for FAD (506.5–581.5 nm) was also statistically significant (p < 0.0001), with two pairwise comparisons being statistically significant (Tumor vs. Muscle: p < 0.0001, Tumor vs. Bone: p = 0.0045, Bone vs. Muscle: p = 0.249). These statistically significant differences were maintained when stratifying tumor into metastatic carcinoma (N = 57) and meningioma (N = 17). TumorID differentiates tumor tissue from normal bone and normal muscle providing further clinical evidence of its efficacy as a tissue identification tool. Future studies should evaluate TumorID’s ability to serve as an adjunctive tool for intraoperative assessment of surgical margins and surgical decision-making.

## Introduction

Spinal tumors represent a heterogenous population of neoplasms categorized as primary or metastatic lesions^1,2^. Surgical resection remains a mainstay of treatment for the majority of spine tumors, with identification of normal versus tumor involved tissue being the most important determinant of recurrence, and for primary tumors, potential cure^3,4^. Extent of resection is often based on interpretation of pre-operative imaging, principles of intraoperative resection, and histopathologic review of the resected tissue. Gross total resection of tumors of the spine is complicated by a breadth of factors pertaining to properties of the neoplasm as well as surgical technique. Subtotal resection is associated with increased rates of local recurrence and suboptimal outcomes, with recurrences occurring more frequently at the margins of resection^5–12^. Intraoperative identification of tumor can be complicated by many factors including visual similarities between healthy tissue and tumor, distorted anatomy from previous procedures, and difficulty identifying the uninvolved tumor margin. Pathology consultations and intraoperative imaging-guided navigation (CT or MRI) are often employed to ensure appropriate tumor resection; however, these techniques may fail to adequately define margin tissue and detrimentally prolong operative times^13–15^.

There is a paucity of studies investigating optical spectroscopic techniques in the neurosurgical spine population^16^. Fluorescence spectroscopy in the form of fluorescence guided surgery has become a promising tool for neurosurgical tumor resection. Current techniques rely upon injectable fluorophores to enhance visualization of neoplastic tissue during surgery and subsequently improve extent of resection^17–19^. Additional avenues for using fluorescence to discriminate tumor from healthy tissue have been explored, but many of these other techniques are complex and expensive^20–22^.

Our group has developed a fluorescence spectroscopy platform, named the TumorID, which distinguishes tumor from healthy tissue based on differences in endogenous fluorophores^23^. The technology utilizes laser-induced non-contact fluorescence spectroscopy to detect tumor cellular metabolic profiles, characterized by distinct concentrations of NADH and FAD from the Warburg effect^24^. Specifically, tissue is excited with a 405 nm laser and the emission fluorescence spectra produced by endogenous fluorophores is analyzed^25^. A 405 nm laser is used to optimally discern differences in NADH and FAD concentrations^26,27^. Previous experiments have demonstrated the technology successfully distinguishes tissue types in murine models^23^. Recent intraoperative ex vivo work with the device demonstrated promise in classifying pituitary adenoma subtypes^28^.

This feasibility study aims to determine whether the TumorID technology can differentiate human clinical specimens ex vivo. Human clinical specimens obtained from patients diagnosed with primary and metastatic spine tumors (healthy normal muscle, healthy normal bone, or tumor) were scanned with the TumorID device and emission spectra were generated to differentiate the tissue types. We validate the TumorID technology as a promising intraoperative tumor identification device for human spine tumor samples.

## Materials and methods

### Study population

This study was conducted under IRB reviewed protocols #00090408 and #00108133. This study was carried out in accordance with relevant guidelines and regulations, samples were obtained under the waiver of consent. Nine adult patients requiring neurosurgical intervention for the management of primary spine tumors or metastatic disease at our institution’s Department of Neurosurgery between November 3, 2022 and February 17, 2023 were identified. Subjects included in this study were confirmed by pathology to have malignancy. Between one and three specimens were obtained per patient: healthy muscle, healthy bone, and/or tumor. The decision to excise either muscle, bone, or tumor was not impacted by this study; tissue was only resected as dictated by the normal course of the procedure (Table 1). Muscle was scanned for patients 3, 4, 6, 7, 8, and 9 (Table 1). Bone was scanned for all patients (Table 1). Tumor was scanned for patients 1, 4, 7, 8, and 9 corresponding with metastatic thyroid carcinoma, metastatic renal cell carcinoma, meningioma, and metastatic breast carcinoma, respectively (Table 1).

**Table 1:**
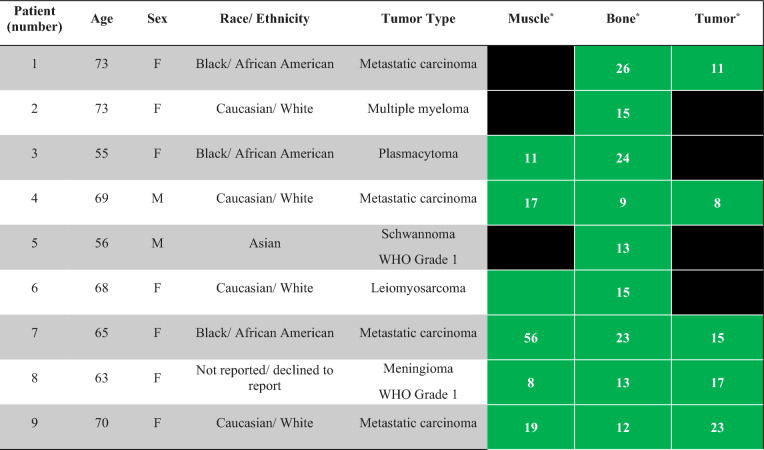
Patient demographics and characteristics.

*Samples of healthy muscle, healthy bone, and tumor were obtained at the discretion of the surgeon. Scans were not obtained for healthy muscle on patients numbered 1, 2, and 5. For patients numbered 2, 3, 5, and 6, tumor was not scanned. Green cells denote that sample was obtained, black cells represent that sample was not obtained. Numbers within the green cells correspond to the number of scans performed.

### Laser specifications

**Figure 1:**
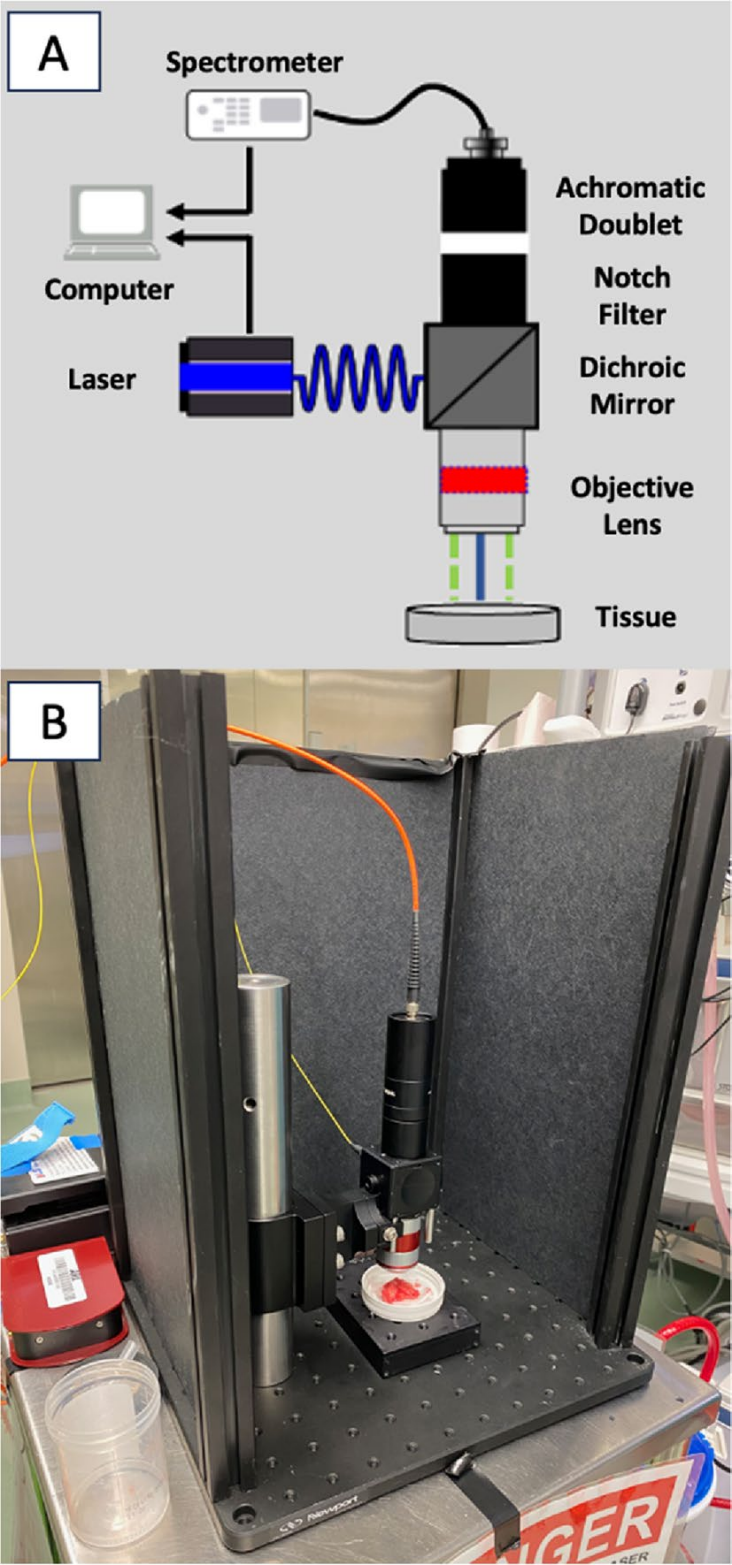
TumorID schematic (**A**) and experimental design (**B**). The laser controller produces the 405 nm light at 100 mW. The light travels through the laser fiber and then is directed through the objective lens by the dichroic mirror. The tumor sample is subsequently irradiated by the light from the objective lens. The emission spectrum is focused by the achromatic doublet as it travels through the spectrometer fiber to the spectrometer. The specimen was positioned beneath the objective lens at a working distance of 17 mm. Each scan was obtained using a wavelength of 405 nm at a power of 100 mW. Prior to each scan, the system was insulated with laser safe material.

Specimens were scanned using a laser induced fluorescence spectroscopy system immediately after resection (Fig. 1). Tissue was positioned 17 mm beneath the objective lens (numerical aperture 0.2, working distance 17 mm, 1/e^2 spot diameter of 0.75 mm) and an excitation wavelength of 405 nm at a power of 100 mW for 0.5 s was used as previously validated^23,29–31^. The system follows a standard epifluorescence design wherein the excitation path and emission path are coupled with a dichroic mirror. The emission was recorded by a CCD spectrometer. Specimens were scanned in at least 5 different locations depending on the size of the tissue; more data was extracted from larger specimens. Tissue was manually positioned and repositioned within the device for each scan. Tissue identity was documented by the surgery and confirmed by pathology after the tissue was scanned with the device.

### Data analysis

The data were first normalized via max normalization. Each scan was first sliced such that only the emission data between 449.0 and 750.2 nm were analyzed. The largest value for each scan was found and then all intensities were normalized by this value, bounding the intensities equal to or less than one. To reduce noise in individual scans, the data were then smoothed by applying a moving average of window size 1.15 nm across the signal. The fluorescence preprocessing utilized is outlined in more detail in previously published work^23,28^. Specific emission spectra corresponding to the fluorophores NADH and FAD were analyzed via calculation of area under the curve (AUC) for representative emission regions. NADH was further stratified into free NADH and bound NADH due distinct emission spectra for NADH depending on its bound state^12,32^. AUC’s were calculated using trapezoidal integration. The bounds of integration were determined by each fluorophore’s emission peak and Full Width at Half Maximum (FWHM) values, specifically [Emission Peak – FWHM/2, Emission Peak + FWHM/2] (Table 2).

**Table 2:** Fluorophore integration bounds^36^.

Fluorophore	Emission peak (nm)	FWHM (nm)	Lower bound (nm)	Upper bound (nm)
Free NADH	487	84	445	529
Bound NADH	501	64	469	533
Free FAD	544	75	506.5	581.5

### Statistical analysis

Researchers operating the TumorID device and statisticians were blinded to the identity of the tissue. Statistical analyses were completed using GraphPad Prism 9.5.3 (GraphPad Software, San Diego, California). For each fluorophore emission region for Bound NADH, Free NADH, and FAD, a Kruskal–Wallis test was conducted comparing the AUC distributions for muscle, bone, and tumor. The use of Kruskal–Wallis was elected after Shapiro–Wilk Test demonstrated that the data did not satisfy normality (Supplementary Table S1). Pairwise comparisons of tissue type were performed using Dunn’s multiple comparisons test. P < 0.05 was used to determine significance.

### IRB approval

This study was carried out under the waiver of consent as approved by the Duke Institutional Review Boards protocol numbers: #00090408 and #00108133. Ethical approval for this study was obtained under IRB reviewed protocols #00090408 and #00108133. All experiments were performed in accordance with the named IRB guidelines and regulations.

## Results

### Demographics

Our study consisted of 9 patients receiving neurosurgical intervention for management of a spine tumor. Seven patients were greater than or equal to 65 years old (Table 1). Seven patients were female and 2 patients were male (Table 1). With respect to race/ethnicity, 4 patients identified as Caucasian/ White, 3 patients identified as Black/African American, 1 patient identified as Asian, and 1 patient declined to report (Table 1). The most common tumor type was metastatic carcinoma consisting of 4 patients, followed by 1 patient with meningioma, 1 patient with multiple myeloma, 1 patient with plasmacytoma, 1 patient with schwannoma, and 1 patient with leiomyosarcoma (Table 1).

### Emission spectra of aggregate tumor, healthy normal muscle, and healthy normal bone

**Figure 2:**
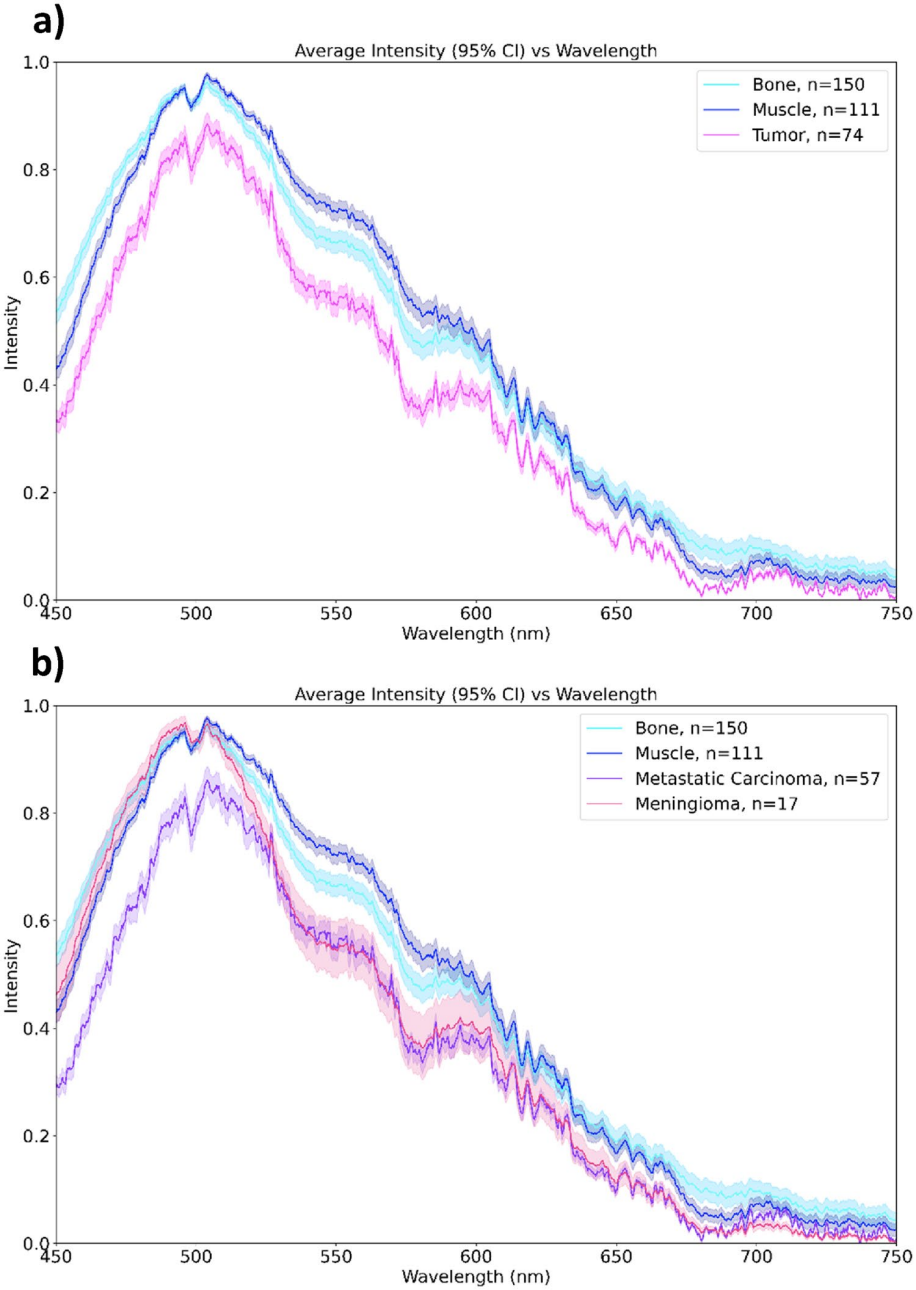
Spectral output comparing (**a**) healthy muscle, healthy bone, and aggregate spine tumor and (**b**) stratifying by tumor type. The x-axis corresponds to emitted wavelength and the y-axis represents normalized intensity. Shading depicts 95% confidence intervals.

**Figure 3:**
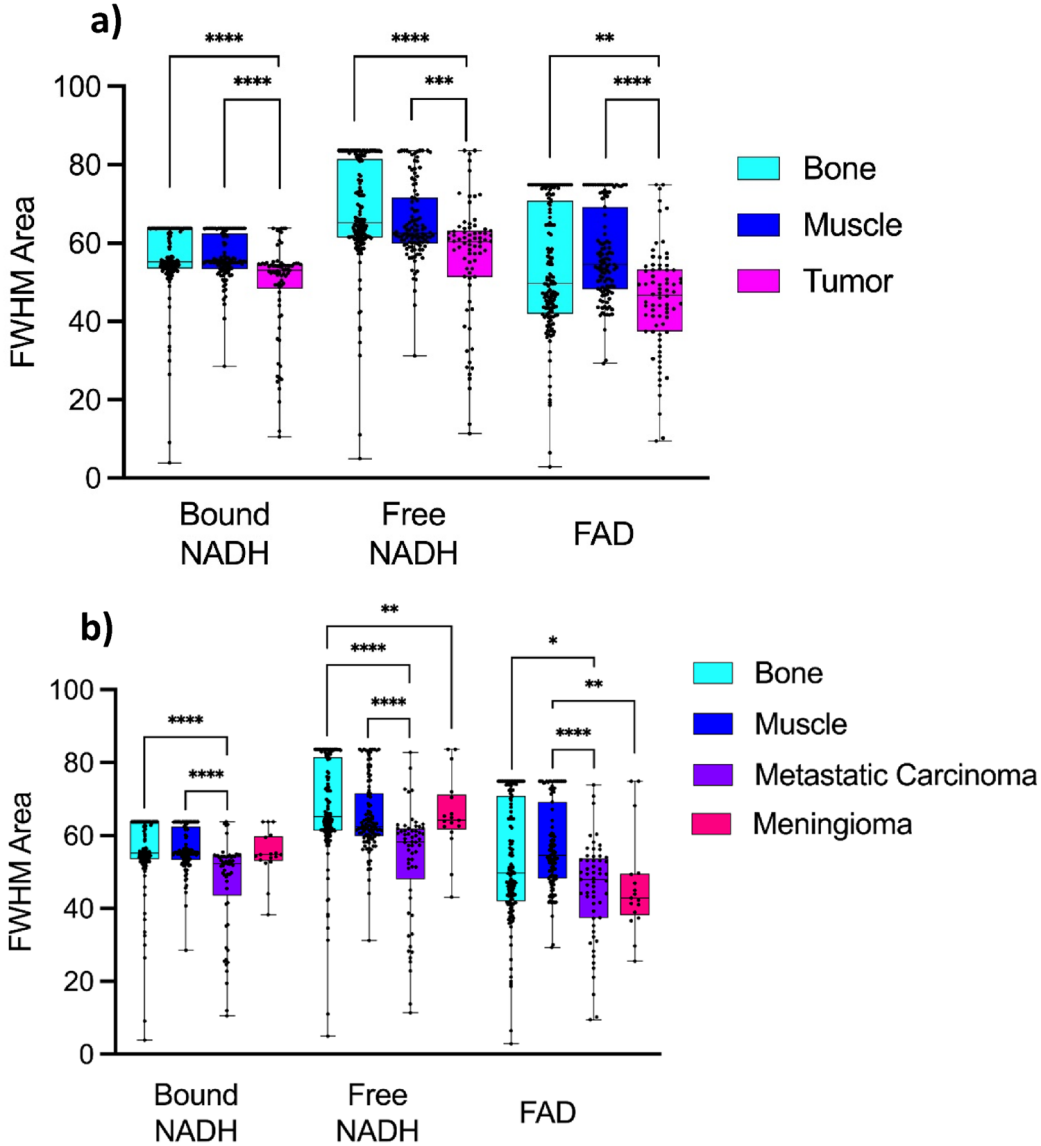
Differences in tissue specific spectra stratified by known emission peaks for FAD, Bound NADH, and Free NADH comparing (**a**) healthy muscle, healthy bone, and aggregate spine tumor and (**b**) stratifying by tumor type. The x-axis represents fluorophore based on known emission of these compounds and the y-axis corresponds to the full width at half maximum (FWHM area). The cyan denotes healthy bone (N = 150), the dark blue represents healthy muscle (N = 111), pink corresponds to tumor (N = 74), purple is metastatic carcinoma (N = 57), and red is meningioma (N = 17). Bars represent minimum and maximum. *p ≤ 0.05, **p ≤ 0.01, ***p ≤ 0.001, ****p ≤ 0.0001.

Emission spectra represents 111 scans of muscle, 150 scans of bone, and 74 scans of tumor (Fig. 2). The spectral output recorded for tumor differed from that of muscle and bone when comparing emission regions of Free NADH, Bound NADH, and FAD (Fig. 2a). In the emission region for Free NADH (445–529 nm) the spectral data of tumor, muscle, and bone when compared were found to be significantly different (Test statistic = 38.02, p < 0.0001) (Table 3, Fig. 3a). Furthermore, all pairwise comparisons between tissue classes were also found to be significantly different (Tumor vs. Muscle: p = 0.0006, Tumor vs. Bone: p < 0.0001, Bone vs. Muscle: p = 0.0357) (Table 3, Fig. 3a). Differences among tissue classes with respect to the emission region for Bound NADH (469–533 nm) was similarly found to be statistically significant (Test statistic = 36.45, p < 0.0001) with two of the three pairwise comparisons being significant (Tumor vs. Muscle: p < 0.0001, Tumor vs. Bone: p < 0.0001, Bone vs. Muscle: p > 0.9999 (ns))^33^. The emission region for FAD (506.5–581.5 nm) was additionally found to be significantly different among tissue types (Test Statistic = 27.13, p < 0.0001) along with all multiple comparisons (Tumor vs. Muscle: p < 0.0001, Tumor vs. Bone: p = 0.0045, Bone vs. Muscle: p = 0.249) (Table 3, Fig. 3a)^33^.

**Table 3:** Kruskal–Wallis test with multiple comparisons for aggregate tumor vs. healthy bone vs. healthy muscle.

Fluorophore	Kruskal–Wallis test		Dunn’s multiple comparison test^1^		
	Test statistic	p	Tumor – muscle: adjusted p value	Tumor—bone: adjusted p value	Muscle—bone: adjusted p value
Bound NADH	36.45	< 0.0001	****	****	ns
Free NADH	38.02	< 0.0001	***	****	*
FAD	27.13	< 0.0001	****	**	*

^1^*p < 0.05, **p ≤ 0.01, ***p ≤ 0.001, ****p ≤ 0.0001.

### Emission spectra of metastatic carcinoma and meningioma, healthy normal muscle, and healthy normal bone

Stratifying tumorous tissues into metastatic carcinoma and meningioma demonstrated statistically significant results for each fluorophore emission region when metastatic carcinoma (N = 57) and meningioma (N = 17) were compared to healthy muscle and healthy bone (Table 4, Fig. 2b).

**Table 4:** Kruskal–Wallis test with multiple comparisons for meningioma vs. healthy bone vs. healthy muscle and metastatic carcinoma vs. healthy bone vs. healthy muscle.

Fluorophore	Kruskal–Wallis test				Dunn’s multiple comparison test^1^			
	Meningioma		Metastatic carcinoma		Meningioma		Metastatic carcinoma	
	Test statistic	p	Test statistic	p	Tumor – muscle: adjusted p value	Tumor—bone: adjusted p value	Tumor – muscle: adjusted p value	Tumor—bone: adjusted p value
Bound NADH	6.32	0.0424	43.35	< 0.0001	ns	ns	****	****
Free NADH	14.15	0.0008	50.23	< 0.0001	ns	**	****	****
FAD	13.87	0.0010	21.72	< 0.0001	**	ns	****	*

^1^*p < 0.05, **p ≤ 0.01, ****p ≤ 0.0001.

For metastatic carcinoma, the emission region for Free NADH was found to be statistically significant when compared to healthy muscle and healthy bone (Test statistic = 50.23, p < 0.0001). For Bound NADH, emission spectra were similarly found to be statistically significant (Test statistic = 43.35, p < 0.0001) and the emission region for FAD was found to be statistically significant (Test statistic = 21.72, p < 0.0001, Table 4, Fig. 3b).

Regarding meningiomas, emission spectra corresponding to Free NADH were statistically significant when compared to healthy muscle and healthy bone (Test statistic = 14.15, p = 0.0008). For Bound NADH, emission spectra were also statistically significant (Test statistic = 6.32, p = 0.0424) and the emission region for FAD was statistically significant (Test statistic = 13.87, p = 0.0010, Table 4, Fig. 3b).

## Discussion

Identification and differentiation of healthy tissue from tumorous tissue is vital for optimizing neurosurgical interventions and subsequent treatment strategies. Fluorescence spectroscopy has been employed in neurosurgery to enhance intracranial resection of diseased tissue; however, these techniques often require administration of exogenous fluorophores which have accompanying side effects^17–19,34–37^. Furthermore, these techniques have had minimal applications in the setting of spine surgery. We sought to investigate whether an internally developed fluorescence spectroscopy device, nominally referred to as the TumorID, was capable of classifying spine tumor, healthy muscle, and healthy bone. We observed differences in the emission spectra corresponding to free NADH, bound NADH, and FAD amongst the three different tissue classes, which remained statistically significant when stratifying tumorous tissue into metastatic carcinoma and meningioma. Tumorous tissue had the lowest intensity spectral signature when compared to healthy muscle and healthy bone. Healthy bone produced a lower intensity spectrum when compared to healthy muscle.

The difference in fluorescence intensity observed across the different tissue types is most likely attributed to the unique metabolic profiles amongst tissue types. Namely, NADH concentrations vary between tumorous tissue and healthy tissue due to increased aerobic glycolysis consuming NADH in highly metabolically active cells^24^. The reduced NADH has an emission profile between 400 and 490 nm whereas the oxidized form of the compound, NAD^+^, does not undergo fluorescence^38^. Therefore, the reduced spectral intensity observed for the tumorous tissue when compared to muscle and bone between 400 and 490 nm may be due to an altered concentration of NADH. The systematic decrease in the signature around 500 is consistent with other publications which demonstrate a similar decrease in emission spectra at 500 nm^33^. Further investigation into the directionality of concentrations of NADH and FAD in distinct tumor types will offer valuable insight into how the technology mechanistically differentiates the tissue. The remainder of the spectrum likely represents contributions from FAD and other endogenously fluorescent compounds. FAD exists in unique oxidation states and degrees of protonation, which are associated with distinct emission spectra; the semi-reduced FAD molecule exhibits an emission spectrum centered around 600-800 nm^39^. Other endogenous fluorophores that would produce emission spectrum between 400 and 700 nm include amino acids, structural proteins, vitamins, and lipids^27^. Differences in emission spectra could further be explained by unique optical properties of the tissue types, with different scattering and absorption coefficients having been reported for glioma, metastasis, meningioma, and glioblastoma; these tissue types scatter and absorb photons in unique manners. Furthermore, oxygenated and deoxygenated hemoglobin are highly absorptive to 405 nm radiation, therefore, recorded spectra are influenced by the quantity of blood present on the specimens^40^.

Fluorescence guided surgery with exogenous fluorophores, such as 5-aminolevulinic acid hydrochloride (5-ALA) and sodium fluorescein (NF), has gained prominence in intracranial neurosurgery. However, these techniques are dependent upon tumorous uptake of fluorescent tracer, which can be dramatically reduced in lower grade gliomas, and requires administration of exogenous compounds that may not be specific for malignant glioma (NF) or have several side effects (5-ALA)^19,34–37^. Furthermore, few studies have looked at potential applications to spine surgery. One recent study demonstrated the use for NF in localizing intradural spinal cord tumors^41^. A case report of two intradural spinal schwannomas describes the use of exogenous indocyanine green dye to more precisely identify tumor margins and discern the lesion from healthy tissue^42^. The literature highlights the potential utility for the TumorID in spine surgery, particularly for resection of intradural tumors. An advantage of the TumorID compared to other fluorescence spectroscopy techniques pertains to the absence of exogenous contrast agents. Thus, time constraints associated with scheduling administration of a dye with the resection as well as potential side effects of the drug are eliminated. Furthermore, the TumorID directly differentiates tissue types based on inherent metabolic differences between tissue types, whereas uptake of exogenous contrast agents serves as an indirect proxy for metabolic activity. The use of label free fluorescence to differentiate tumor types has been described by Marcu and colleagues with Fluorescence Lifetime Imaging (FLIM), and although FLIM is a powerful tool, it has limitations^43^. FLIM measures the duration that a fluorophore remains in an excited state, compared to most fluorescence spectroscopy techniques that characterize the intensity and spatial distribution of a signal^44^. FLIM offers the advantage of a high resolution snapshot of cellular biochemistry, but is limited by acquisition speeds of up to one minute per scan as well as complexity and cost of the systyem^43,45,46^. TumorID utilizes less technically complex steady-state intensity-based fluorescence spectroscopy compared to FLIM, which significantly increases the speed at which scans can be conducted and reduces both associated cost and complexity while still retaining clinical utility.

The TumorID remains an exciting methodology for discerning tumor from healthy tissue, however, there are several limitations to this work. Bone displayed a high level of reflectivity to the incident 405 nm radiation which presented challenges to data collection that have been described previously^47^. This is not an unexpected result as the difference in color as well as the frequent lack of blood and hemoglobin products on the surface would allow for much greater reflectivity. The increased reflectivity frequently caused overexposure of the spectrometer, and therefore spectral recordings that could not be used for statistical analysis. The incident power would be decreased until a correctly exposed scan was captured, however this means that less incident radiation produced these results. The observation that bone responds differently to the same incident radiation is a powerful discovery for future classification work. Furthermore, blood and blood breakdown products, such as porphyrins and heme, hinder analyses as both oxygenated hemoglobin and deoxygenated hemoglobin are strong absorbers of 405 nm radiation^48^. Resultant scans are of lower intensity when large quantities of blood are present on the surface. Additionally, while beneficial that the device does not contact tissue, this also requires exposure of the tissue that is being analyzed to incident radiation. As with all lasers, there is inherent risk of burning tissue, although the device has been shown to not induce thermal tissue after rigorous testing in collaboration with our institution’s pathology department.

While this study focused on the use of the TumorID in detecting neoplastic spine lesions, there remain several exciting implications and applications of the device in intracranial neurosurgery. Fluorescence spectroscopy is often employed to enhance reliability and accuracy of intracranial tumor resections. However, these techniques would benefit from an accurate tumor detecting modality that does not rely upon both administration and uptake of exogenous contrast agents^17–19,34–37^. Future applications of this technology could potentially include aid in identification of micrometastases or other malignant tissue not detected by current imaging techniques. Outside of fluorescence spectroscopy, neuronavigation is often used to aid in the identification of tumorous tissue, but a common issue faced by surgeons is brain shift or slight movement of fiducials, which can result in inaccurate tumor localization^15^. The TumorID real time fluorescence technology provides an adjunctive tool to reduce error associated with misaligned neuronavigation. Furthermore, the device precisely analyzes small pieces of tissue with diameter 0.75 mm with minimal tissue penetration with the potential to communicate to the physician the identity of the tissue that is being visualized.

The results from this study warrant future investigation utilizing the TumorID. Validity of the device as an intraoperative tool can be pursued with in vivo studies assessing the replicability of the results from this ex vivo study. Previous work from our group has demonstrated potential to classify pituitary adenoma subtypes and additional experimentation assessing the ability of the device to identify spine and intracranial tumor types would yield valuable information^28^. Use of the device in the pathology setting might confer increased reliability and confidence in diagnoses. Furthermore, analyses of non-tumor pathology would also serve an interesting area for exploration (Supplementary Table S1).

## Conclusion

Differentiation of tumorous tissue from healthy tissue is vital to resect neurosurgical tumors safely and effectively. Current techniques for distinguishing tumor from surrounding tissue rely upon administration of exogenous agents or neuronavigational technologies, both of which have inherent shortcomings. This work demonstrated that the TumorID can successfully differentiate spine tumor from healthy muscle and healthy bone utilizing non-contact endogenous fluorescence spectroscopy. The emission spectra of tumorous tissue had a diminished normalized intensity when compared to healthy muscle and bone in regions corresponding to Free NADH, Bound NADH, and FAD. Future studies will be valuable in establishing the validity of the device as an intraoperative tool and further exploring the classification capacity of the technology.

### Supplementary Information

Supplementary Table S1.

## Data Availability

The datasets used and/or analyzed during the current study available from the corresponding author on reasonable request.
