# Supplementary material for: A blinded study using laser induced endogenous fluorescence spectroscopy to differentiate ex vivo spine tumor, healthy muscle, and healthy bone
Source: Sci Rep. 2024 Jan 22;14:1921. doi: 10.1038/s41598-023-50995-4 (PMC10803777; doi:10.1038/s41598-023-50995-4)
Supplement: Supplementary file 1 — Supplementary Table S1. [file 41598_2023_50995_MOESM1_ESM.docx]

**Supplemental Tables**

**Table S1. Shapiro-Wilk Normality Test**

|  | **Bone** | | | **Muscle** | | | **Tumor** | | |
| --- | --- | --- | --- | --- | --- | --- | --- | --- | --- |
|  | **Bound NADH** | **Free NADH** | **FAD** | **Bound NADH** | **Free NADH** | **FAD** | **Bound NADH** | **Free NADH** | **FAD** |
| **W** | 0.6946 | 0.8493 | 0.9225 | 0.8673 | 0.9156 | 0.9179 | 0.8002 | 0.8983 | 0.9738 |
| **p Value** | <0.0001 | <0.0001 | <0.0001 | <0.0001 | <0.0001 | <0.0001 | <0.0001 | <0.0001 | 0.1276 |
| **Normal?** | No | No | No | No | No | No | No | No | Yes |
| **Significance** | **** | **** | **** | **** | **** | **** | **** | **** | ns |

****p ≤ 0.0001.
